# Dual-energy X-ray absorptiometry: an effective approach for predicting broiler chicken body composition

**DOI:** 10.1016/j.psj.2023.103363

**Published:** 2023-12-09

**Authors:** Gustavo A.C.C. de Aguiar, Lucimauro da Fonseca, Maria R.S. de Farias, Gabriel R. Braga, Joyce Barcellos, Érica B. Schultz, Melissa I. Hannas

**Affiliations:** Department of Animal Science, Universidade Federal de Viçosa, 36570-900 Viçosa, MG, Brazil

**Keywords:** DEXA prediction, chicken, carcass body composition, chemical analysis, validation

## Abstract

Two trials were carried out to develop and validate linear regression equations for body composition prediction using Dual-energy X-ray absorptiometry (**DEXA**). In Trial 1, 300 Cobb500 male chickens raised from 1 to 42 d of age were scanned in DEXA to estimate total weight, fat mass, soft lean tissue (**SLT**) mass, bone mineral content (**BMC**), and fat percentage. DEXA estimates were compared to body ash, crude fat, SLT (sum of protein and water) and scale body weight. The dataset was split, with 70% used for prediction equations development and 30% for testing, and the 5k-fold cross-validation analysis was used to optimize the equations. The R^2^, mean absolute error (**MAE**), and root-mean-squared error (**RMSE**) were used as precision and accuracy indicators. A negative correlation (*ρ* = ˗0.27) was observed for ash content, while no correlation was observed for protein content (*P* > 0.05). Predictive linear equations were developed to assess broiler weight (R^2^ = 0.999, MAE = 25.12, RMSE = 38.99), fat mass (R^2^ = 0.981, MAE = 13.87, RMSE = 21.28), ash mass (R^2^ = 0.956, MAE = 3.98, RMSE = 5.61), SLT mass (R^2^ = 0.997, MAE = 35.73, RMSE = 52.45), water mass (R^2^ = 0.997, MAE = 29.56, RMSE = 43.94), protein mass (R^2^ = 0.989, MAE = 12.94, RMSE = 19.05), fat content (R^2^ = 0.855, MAE = 0.81, RMSE = 1.05), SLT content (R^2^ = 0.658, MAE = 1.01, RMSE = 1.28), and water content (R^2^ = 0.678, MAE = 0.99, RMSE = 1.27). All equations passed the test. In Trial 2, 395 Cobb500 male chickens were raised from 1 to 42 d of age and used for validation of prediction equations. The equations developed for weight, fat mass, ash mass, SLT mass, water mass, and protein mass were validated. In conclusion, DEXA was found to be an effective approach for measuring the body composition of broilers when using predictive equations validated in this study for estimate calibration.

## INTRODUCTION

Over the years, the poultry industry has undergone changes to meet increasing demand for meat from the global population and minimize economic losses. Furthermore, in commercially-raised birds, such as broilers and laying hens, understanding body composition is valuable for various purposes, including breeding and selection programs, nutritional recommendations, and poultry management. Birds with fast growth have been successfully selected to achieve target body weight earlier and for feed efficiency (Havenstein et al., 2003). However, these selection programs have led to several indirect consequences, such as metabolic disorders, leg problems, and increased fat deposition (Shim et al., 2012; Schallier et al., 2019). In order to detect these inherent problems and help breeders take appropriate actions, methods for measuring chickens' body compositions have been employed. Carcass chemical analysis is the most commonly used method to assess body composition and is considered the gold standard for this process. However, this method is time-consuming, complex, invasive, and requires the animal to be sacrificed to establish the content of protein, fat, water, and ash (Kasper et al., 2021; Martinez et al., 2022b). Although carcass chemical analysis produces reliable measurements, it does not allow for repeated measurements on the same animal throughout its growth, which is feasible with live animal methods. Furthermore, live animal methods meet the ethical demands of certain groups of society for animal care and use, by preventing slaughter of several individuals; moreover, this method can reduce costs and environmental pollution associated with chemical analysis (Gonçalves et al., 2018).

Dual-energy X-ray absorptiometry (**DEXA**) is a noninvasive method of measuring body composition that was originally designed for humans, but in recent decades, has been used as a tool in livestock research (Scholz et al., 2015), and in the food industry for meat quality inspection (Pipe, 2023). The equipment software uses the reduction of the dual X-ray beam caused by distinct absorbing materials to estimate the values of total tissue, soft lean tissue (**SLT**), fat tissue, bone mineral content (**BMC**), and fat percentage in the projected figures (Schallier et al., 2019). DEXA has been proven useful in animal science; its application varies between prediction of intramuscular fat in beef steak (Nunes et al., 2023), body composition of swine (Soladoye et al., 2016; Kasper et al., 2021), body composition and phosphorus retention in rainbow trout (Ndiaye et al., 2020), mineral density and content in laying hens (Hester et al., 2004; Schreiweis et al., 2004), broiler bone resistance (Shim et al., 2012), and growth performance (Castro et al., 2019; Meyer et al., 2019). Furthermore, DEXA is a promising noninvasive method for longitudinal studies to determine fast heating production, and consequently net energy requirements of chicken growth (Martinez et al., 2022a; Suesuttajit et al., 2022), and longitudinal studies about potential body growth of broiler and pullet strains (Alves et al., 2019; Gonçalves et al., 2020).

Despite these advantages, DEXA needs to be calibrated to estimate the body composition of birds because the technique is not capable of directly estimating protein and water contents. Some works have shown that DEXA is effective at estimating body composition of chickens by comparing DEXA results with chemical analysis (Mitchell et al., 1997; Swennen et al., 2004; Salas et al., 2012; Gonçalves et al., 2018; Schallier et al., 2019). According to Mitchell et al. (2011), chicken feathers are not detected by DEXA. In this sense, the DEXA results must be related to chemical composition of the feather-free body (**FFB**) of broilers (Alves et al., 2019). To the best of our knowledge, Schallier et al. (2019) is the most recent study to use DEXA to predict body composition of chickens, for which the authors developed reliable prediction regressions; however, in that study, the birds’ ages ranged from 21 to 56 d. Nonetheless, there are different brands of DEXA devices and they are all equipped with specific software versions, necessitating the development of individual prediction regression models for all DEXA devices and software combinations (Swennen et al., 2004; Kasper et al., 2021).

For this reason, 2 trials were carried out in the present study. Trial 1 aimed to develop appropriate prediction equations for broiler body composition using a Lunar Prodigy Advance DEXA fan beam scanner (software enCORE v18SP2) and chemical analysis, with broilers of different body compositions ranging from 1 to 42 d of age. Trial 2 aimed to externally validate the models developed to predict the body composition of broilers obtained in Trial 1.

## MATERIALS AND METHODS

### Ethics

Experiments were conducted to create regression equations (Trial 1) and for equation validation (Trial 2). All procedures adopted for both trials were previously approved by the Ethics Committee in the Use of Farming Animals at the Universidade Federal de Viçosa (**CEUAP-UFV**) under the accession number 029/2020 for Trial 1 and the accession number 057/2022 for Trial 2.

### DEXA Scanning

**Figure 1:**
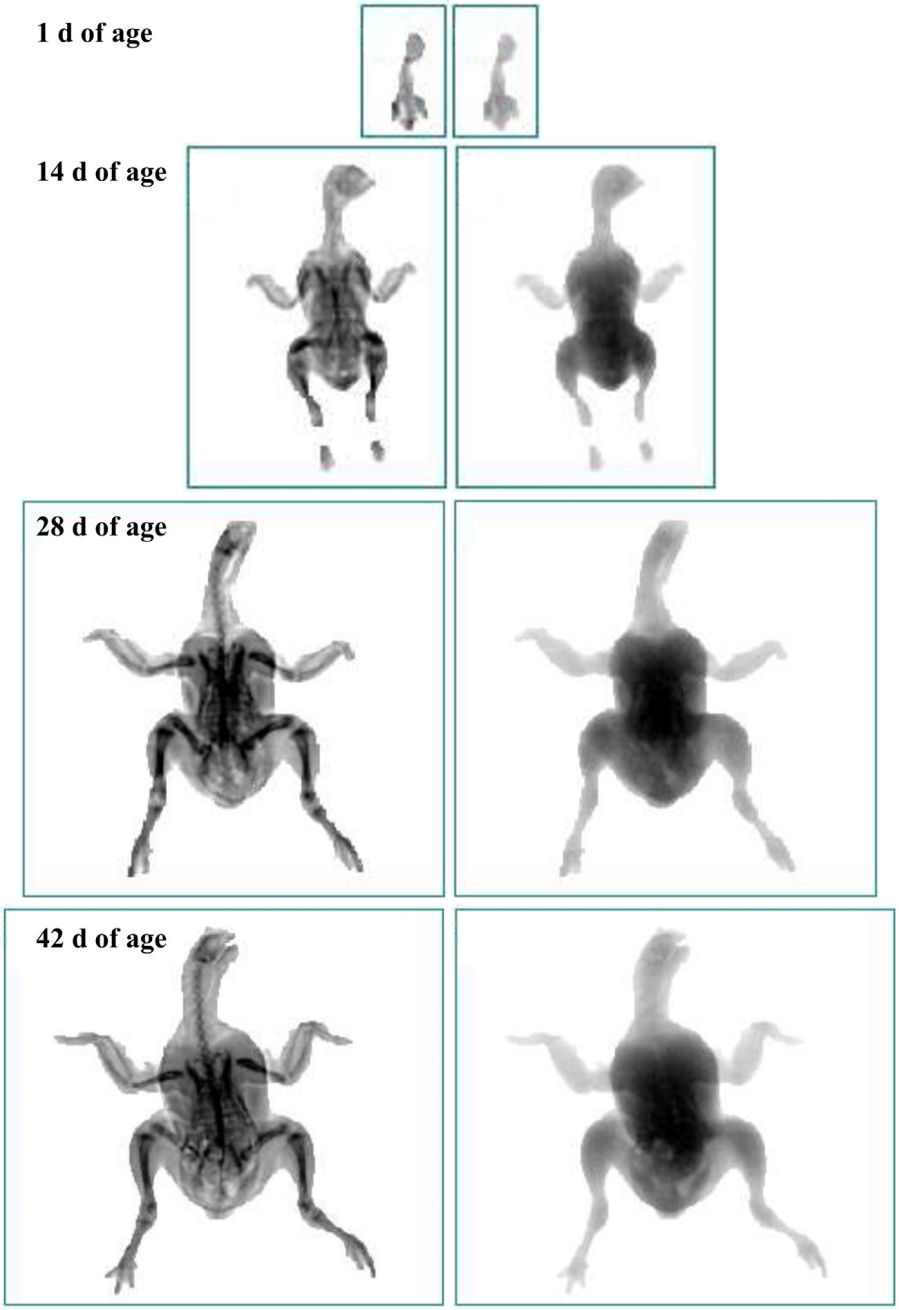
DEXA scan image of a broiler. Square lines define the region of interest (ROI) applied to starter, grower and finisher groups of scanned birds.

Birds’ body composition was measured using the standard mode of the small animal module of the DEXA scanner model Lunar Prodigy Advance (PRODIGY, GE HEALTHCARE, Madison, WI) equipped with enCORE v18SP2 software in Laboratory of Body Composition and Densitometry of the Department of Animal Science of the Universidade Federal de Viçosa (**UFV**). In each day prior to the scanner analyses of the birds, a quality assurance (**QA**) program was performed using a phantom standard to ensure an accurate calibration of the equipment. Next, the birds were placed in the dorsal position on the scanner table with spread wings and stretched legs to avoid overlap of body parts. Birds were scanned from the cranial to caudal direction. After the scanning, the “region of interest” (**ROI**) was selected and personalized by drawing a custom rectangular area around the whole body of the bird image, as shown in Figure 1. The following DEXA traits were recorded: SLT (g), fat tissue (g), BMC (g), and fat percentage (%). The sum of soft lean and fat tissue with the BMC of the DEXA measurements was assumed to be the total body weight (g). SLT (%) and BMC (%) were calculated using the respective gram values as a ratio of total body weight.

### Trial 1: Development of Linear Regression Equations

For regression equation development a total of 300 Cobb500 male broilers were raised from 1 to 42 d of age. Diets were formulated for starter (1–14 d of age), grower (14–28 d of age), and finisher (28–42 d of age) feeding phases according to Rostagno et al. (2017) (as shown in Supplemental Table S1), with various levels of methionine inclusion. The application of these ages and diet variations were important for achieving a wide range of body weights and compositions, which is essential for establishing accurate regression equations. During the experimental period, birds were reared on floor pens under standard temperature and light conditions according to genetics guidelines, with diets and water provided ad libitum. Nine 1-day-old birds and 97 birds for each age group of 14, 28, and 42 d of age were selected, respectively, for a fasting period of 12 h, and then euthanized by cervical dislocation and defeathered. As DEXA does not detect feathers, we chose to pluck and freeze carcasses prior to scanning to facilitate future processing for chemical analysis, and to allow scanning of several number of birds at the same age. The whole carcasses FFB (including organs, heads, and limbs) were weighted, then frozen (−20°C) and later defrosted for DEXA scanning analysis according to the aforementioned method.

After scanning, each of the FFB carcasses were frosted again and were further cut with a band saw (SI-282HD, SKYMSEN, Volta Grande, SC, Brazil), grounded twice with industrial grinder (PBM081, BECCARO, Rio Claro, SP, Brazil), homogenized, and a sample was collected to be lyophilized for chemical analysis conducted at the Laboratory of Animal Nutrition of the Department of Animal Science of the UFV. One-day-old birds were pooled before lyophilization to obtain 3 representative samples. Lyophilized samples were ground in a ball mill and used for analysis of dry matter (INCT-CA, 2021), crude protein Kjeldahl N × 6.25 (INCT-CA, 2021), ether extract (AOCS, Am 5-04, 2009), and ash (INCT-CA, 2021). All chemical values obtained were converted into natural matter to express the quantitative amount of total ash, ether extract, and the protein of total FFB composition (with water) in order to be compared with DEXA scan results. Regarding the 1-day-old birds DEXA scan results, the mean of 3 chicks used for the pool was compared with the wet chemical counterpart (total *n* = 294). Due to the fact that DEXA SLT mass considers the amount of protein and water of tissue, the chemical SLT was considered the sum of crude protein and water.

### Trial 2: Linear Regression Equations Validation

A second trial was conducted to evaluate the accuracy of the regression equations developed in Trial 1. A total of 395 Cobb500 male broilers were raised from 1 to 42 d of age on floor pens. During the rearing period, the animals received corn and soybean-meal based diets in order to meet or exceed the requirements established by Rostagno et al. (2017) from 1 to 21 and from 22 to 42 d of age, with variations in mineral sources and quantities (shown in Supplemental Table S2). Birds received diet and water ad libitum and the ambient temperature and the light program were controlled following the genetics guidelines. At the beginning of Trial 2, a group of 10 one-day-old birds were selected, fasted 12 h, and euthanized by cervical dislocation. In order to acquire sufficient data from chickens with different ages for validation of the prediction equations developed in Trial 1, at the end of each 14, 21, 28, 35 and 42 d of rearing, a total of 77 birds were selected, fasted 12 h, euthanized, defeathered, and frozen, respectively. Afterward, the FFB broiler carcasses were defrosted and the FFB carcasses were scanned using the DEXA method mentioned previously, and then all FFB carcasses were frozen for further chemical analysis procedures like those described in Trial 1. For all body composition traits, 77 chickens per age group (14, 21, 28, 35, and 42 d of age) were used for chemical analyses of protein, water, and ash (total *n* = 385). However, 55 chickens for each age groups, were used for fat traits (total *n* = 275). Ten 1-day-old birds were not processed for chemical analysis, a decision that is explained below.

### Statistical Analysis

DEXA measurements and wet chemical data were organized into the following variables: weight, fat, ash, SLT, water, and protein of the FFB broiler expressed as weight (g) and content (%) (Table 1). All statistical procedures were performed using R software (R Core Team, 2022), the script of which is available in the supplemental material (Table S3). In all statistical tests described below, results were considered significant when *P* < 0.05.

**Table 1:** Data divided in age groups of Trial 1 used for development of linear regression equations, and of Trial 2 used for validation. DEXA estimates, chemical analysis and predicted values are presented in means, and in parenthesis the coefficient of variation (%).

	Trial 1^1^			Trial 2^2^		
Item	DEXA	Chemical	Predicted	DEXA	Chemical	Predicted
1 day old						
Weight, g	30.88 (1.25)	44.44 (0.43)		36.51 (1.91)		55.67 (1.35)
Fat, %	5.41 (39.34)	5.58 (13.59)		11.34 (16.09)		9.65 (8.93)
Fat, g	1.67 (40)	2.48 (13.24)		4.14 (16.66)		14.95 (3.49)
Ash, %	0.32 (1.26)	2 (4.28)				
Ash, g	0.1 (0)	0.89 (3.88)		0.37 (13.14)		7.73 (1)
SLT^3^, %	94.29 (2.23)	91.44 (1.56)		87.64 (2.18)		86.86 (0.68)
Water, %		75.89 (1.5)				71.16 (0.86)
Protein, %		15.55 (4.8)				
SLT^3^, g	29.11 (1.32)	40.64 (1.99)		32 (2.55)		5.22 (18.11)
Water, g		33.73 (1.91)				7.14 (10.75)
Protein, g		6.91 (5.02)				−1.94 (9.14)
14 days old						
Weight, g	434.2 (7.32)	481.2 (6.67)	481.5 (6.99)	500.7 (7.94)	547.5 (7.34)	556.5 (7.71)
Fat, %	7.06 (23.12)	7.81 (14.35)	7.65 (12.17)	10.03 (14.82)	7.69 (16.78)	9.09 (8.02)
Fat, g	30.53 (22.49)	37.59 (16.5)	34.77 (16.84)	50.38 (18.03)	42.24 (20.43)	50.33 (14.1)
Ash, %	1 (11.38)	3.02 (11.08)				
Ash, g	4.33 (12.58)	14.53 (12.21)	14.23 (5.46)	5.66 (11.87)	13.45 (15.24)	16.07 (6.59)
SLT^3^, %	91.94 (1.8)	87.86 (1.53)	88.16 (0.69)	88.84 (1.69)	88.65 (1.35)	87.23 (0.53)
Water, %		72.3 (2.12)	72.52 (0.87)		74.48 (1.35)	71.55 (0.68)
Protein, %		15.56 (5.08)				
SLT^3^, g	399.3 (7.89)	422.8 (6.81)	426.8 (8.86)	444.7 (7.64)	485.2 (7.23)	482.7 (8.14)
Water, g		347.8 (6.56)	349.8 (8.79)		407.7 (7.22)	395.1 (8.08)
Protein, g		74.96 (9.68)	77.05 (9.2)		77.53 (8.95)	87.49 (8.41)
21 days old						
Weight, g				1069 (6.55)	1141 (5.89)	1170 (6.46)
Fat, %				10.35 (14.77)	7.93 (17.22)	9.21 (7.83)
Fat, g				110.7 (16.12)	90.4 (17.21)	95.89 (14.12)
Ash, %						
Ash, g				13.59 (9.05)	27.95 (12.07)	28.58 (6.79)
SLT^3^, %				88.38 (1.72)	88.84 (1.13)	87.09 (0.54)
Water, %					73.99 (1.38)	71.4 (0.68)
Protein, %						
SLT^3^, g				944.9 (6.75)	1013 (6.09)	1062 (6.95)
Water, g					844.1 (6.26)	865.3 (6.93)
Protein, g					169.4 (6.31)	195.9 (7.05)
28 days old						
Weight, g	1395 (5.24)	1525 (4.93)	1519 (5.49)	1700 (6.52)	1785 (6.3)	1850 (6.47)
Fat, %	16.1 (14.61)	11.36 (11.7)	11.82 (10.32)	12.04 (12.34)	9.85 (16.27)	10.01 (6.82)
Fat, g	223.9 (13.18)	172.9 (10.7)	178.9 (12.89)	204.74 (14.28)	175.46 (17.26)	167.1 (12.87)
Ash, %	1.6 (9.66)	2.71 (9.58)				
Ash, g	22.3 (8.29)	41.42 (10.91)	42.38 (6.4)	22.31 (9.98)	42.43 (12.11)	42.35 (8.29)
SLT^3^, %	82.29 (2.95)	85.28 (1.78)	85.26 (0.96)	86.65 (1.71)	87.95 (1.3)	86.55 (0.53)
Water, %		69.34 (1.92)	69.49 (1.23)		72.85 (1.63)	70.84 (0.67)
Protein, %		15.95 (5.03)				
SLT^3^, g	1149 (6.96)	1301 (5.91)	1299 (7.97)	1473 (6.56)	1570 (6.49)	1672 (6.69)
Water, g		1058 (5.76)	1058 (7.94)		1300 (6.56)	1361 (6.67)
Protein, g		243.5 (8.15)	240.3 (8.06)		269.6 (7.76)	310.3 (6.75)
35 days old						
Weight, g				2475 (6.85)	2582 (6.7)	2687 (6.81)
Fat, %				11.39 (16.46)	10.26 (15.57)	9.54 (9.07)
Fat, g				281.3 (18.79)	264.7 (17.67)	219.6 (18.13)
Ash, %						
Ash, g				29.54 (8.46)	59.51 (9.72)	53.75 (7.34)
SLT^3^, %				87.47 (2.1)	87.29 (1.33)	86.81 (0.65)
Water, %					72.25 (1.81)	71.1 (0.83)
Protein, %						
SLT^3^, g				2164 (6.69)	2253 (6.77)	2472 (6.78)
Water, g					1865 (7.12)	2012 (6.77)
Protein, g					388.1 (6.46)	460.1 (6.82)
42 days old						
Weight, g	2800 (3.28)	3034 (2.84)	3035 (3.84)	3091 (7.71)	3240 (7.62)	3352 (7.68)
Fat, %	18 (14.1)	13.05 (14.65)	12.78 (7.6)	12.94 (17.62)	11.06 (15.26)	10.45 (9.81)
Fat, g	503.6 (13.93)	395.4 (14.17)	391.6 (10.69)	401.1 (20)	361.8 (17.57)	318.9 (17.6)
Ash, %	1.53 (8.34)	2.48 (11.19)				
Ash, g	42.81 (7.94)	75.2 (11.8)	74.98 (7.62)	38.37 (10.33)	75.7 (10.88)	67.69 (9.24)
SLT^3^, %	80.47 (3.16)	85 (2.2)	84.65 (0.74)	85.82 (2.63)	86.76 (1.6)	86.3 (0.81)
Water, %		69.2 (2.5)	68.86 (0.95)		71.86 (2.05)	70.57 (1.03)
Protein, %		15.8 (5.23)				
SLT^3^, g	2254 (4.95)	2579 (4.02)	2575 (5.32)	2652 (7.72)	2810 (7.56)	3036 (7.8)
Water, g		2099 (4.14)	2095 (5.31)		2328 (7.75)	2470 (7.79)
Protein, g		479.4 (6.4)	479.4 (5.35)		482.4 (8.38)	565.8 (7.84)
1–42 days old						
Weight, g	1517 (64.88)	1651 (64.23)	1572 (64)	1709 (57.06)	1798 (56.21)	1860 (56.56)
Fat, %	13.57 (39.24)	10.66 (25.23)	10.64 (23.1)	11.31 (18.11)	9.29 (21.59)	9.64 (9.86)
Fat, g	247.9 (80.77)	198.3 (76.82)	186.9 (76.27)	202.5 (66.7)	181.4 (67.5)	162.1 (62.74)
Ash, %	1.36 (23.35)	2.73 (13.67)		1.22 (9.66)		
Ash, g	22.72 (70.52)	42.98 (60.03)	41.5 (58.05)	21.15 (57.48)	42.39 (55.47)	40.52 (47.35)
SLT^3^, %	85.07 (6.57)	86.12 (2.45)	86.1 (1.95)	87.47 (2.35)	87.97 (1.67)	86.81 (0.73)
Water, %		70.4 (3.09)	70.37 (2.49)		73.23 (2.4)	71.1 (0.93)
Protein, %		15.76 (5.19)				
SLT^3^, g	1246 (62.15)	1410 (63.67)	1344 (63.19)	1485 (56.23)	1574 (55.49)	1687 (57.29)
Water, g		1149 (63.52)	1095 (63.03)		1305 (55.26)	1373 (57.17)
Protein, g		261.3 (64.63)	248.8 (63.93)		268.2 (56.86)	313 (57.82)

1 *n* = 294.

2 *n* = 385; except fat chemical analysis (*n* = 275).

3 SLT: soft lean tissue.

The first step (*n* = 294) for the development of regression equations for each variable was to verify the confidence interval (**CI**) and significance of the least-squares linear regression parameters: intercept (**β_0_**) and slope (**β_1_**). Afterward, a Pearson correlation (**ρ**) analysis between DEXA measurements and wet chemical data was performed.

The second step was to select, based on *β*_0_, *β*_1_, and *ρ* significances, what body composition variables were able for development of the linear regressions. Therefore, to verify that the model was not overfitted, a random split was performed on the Trial 1 dataset for a 5k-fold cross-validation analysis; 70% of dataset (*n* = 206) was used for training to create the linear regression equations, and the remaining 30% (*n* = 88) was used to test the prediction of linear regressions. Additionally, the mean absolute error (**MAE**) and the root-mean-squared error (**RMSE**) was estimated for each variable, and together with the coefficient of determination (**R^2^**), were used as indicators of precision and accuracy.

The third step was to validate the linear regression equations created and tested with Trial 1 dataset using the Trial 2 dataset. DEXA measurements of Trial 2 were used in linear regression equations to obtain the predicted body composition values. The 1-day-old birds from Trial 2 data were not used for validation because the prediction results were not reliable (e.g., negative values observed for protein mass) (Table 1). The predicted values were then compared to the wet chemical counterparts with a regression analysis (predicted vs. observed) and R^2^, MAE, RMSE, and *P* were assessed as precision and accuracy indices (*n* = 385, except fat = 275).

## RESULTS

### Variation in Body Composition of Broiler Chickens

Variation in body composition of broiler chickens of different ages for Trial 1 and Trial 2 was achieved; the means with the coefficient of variation are shown in Table 1. Regardless of birds’ ages or analysis method (DEXA or chemical), and in both trials, body measurements from highest to lowest variation were: fat, ash, protein, weight, SLT, and water.

### Trial 1: Development of Linear Regression Equations

The linear regression parameters of variables measured by DEXA scanner and chemical carcass analysis are presented in Table 2. *β*_0_ indicates the intercept and *β*_1_ the slope of linear regression, and their respective CI are also exhibited. The degree of correlation is indicated by *ρ* as the correlation coefficient and the R^2^ are also shown in this table.

**Table 2:** Linear regression parameters (*β*_0_ and *β*_1_) and confidence interval (CI) of broilers body composition DEXA vs. chemical analysis. Birds aging 1 to 42-day old. Correlation coefficient (*ρ*) and coefficient of determination (R^2^) are shown (*n* = 294).

	Intercept			Slope				
Item	*β*_0_	CI	*P*	*β*_1_	CI	*P*	R^2^	*ρ*
Weight, g	17.33	9.48–25.18	<0.001	1.08	1.07–1.08	<0.001	0.999	1*
Fat, %	4.40	4.06–4.75	<0.001	0.46	0.44–0.49	<0.001	0.833	0.91*
Fat, g	11.45	7.66–15.25	<0.001	0.75	0.74–0.77	<0.001	0.982	0.99*
Ash, %	3.16	2.98–3.34	<0.001	−0.31	−0.44 to −0.18	<0.001	0.070	−0.27*
Ash, g	7.28	6.15–8.40	<0.001	1.57	1.53–1.61	<0.001	0.953	0.98*
SLT^1^, %	60.67	58.41–62.93	<0.001	0.30	0.27–0.33	<0.001	0.628	0.79*
Water, %	44.01	41.71–46.31	<0.001	0.31	0.28–0.34	<0.001	0.635	0.80*
Protein, %	16.66	15.23–18.09	<0.001	−0.01	−0.03 to 0.01	0.22	0.005	−0.07^NS^
SLT^1^, g	−32.14	−42.97 to −21.31	<0.001	1.16	1.15–1.17	<0.001	0.997	1*
Water, g	−23.23	−32.49 to −13.97	<0.001	0.94	0.93–0.95	<0.001	0.997	1*
Protein, g	−8.90	−12.79 to −5.01	<0.001	0.22	0.21–0.22	<0.001	0.989	0.99*

1 SLT: soft lean tissue.

⁎ Correlation is significant with *P* < 0.001.

NS Correlation is not significant (*P* > 0.05).

**Figure 2:**
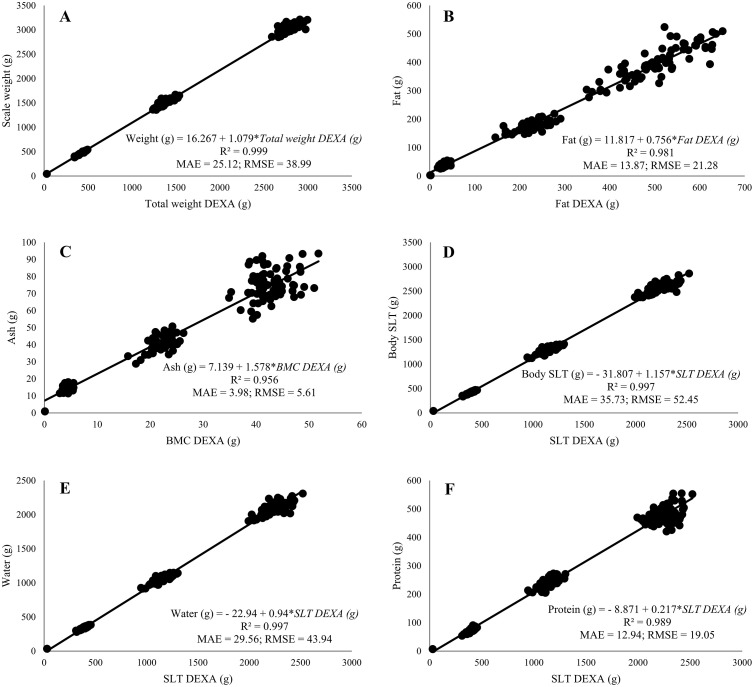
Least-squares regressions developed using 70% of Trial 1 dataset for prediction of weights of broiler body composition: weight (A), fat (B), ash (C), soft lean tissue (SLT) (D), water (E), and protein (F). The DEXA measurements are independent variables on *x*-axis and corresponding chemical analysis values are dependent variables on *y*-axis. Regression equations, coefficients of determination (R^2^), mean absolute error (MAE) and the root-mean-squared error (RMSE) are shown in every graphic (*n* = 206).

The total weight measured by DEXA scanner was the sum of SLT, fat, and BMC tissues and was compared with the carcasses scale weight. For weight mass linear regression parameters verification, both *β*_0_ and *β*_1_ were significant (*P* < 0.001) and a high positive correlation (*ρ* = 1) and R^2^ value (0.999) between DEXA weight and scale weight was observed (Table 2). Based on these results, a linear regression equation was developed to estimate the broiler carcass weight (R^2^ = 0.999, MAE = 25.12, RMSE = 38.99) and is shown in Figure 2A. The test of regression equations of the weight mass with Trial 1 data split is shown in Table 3, and a high R^2^ (0.999) value was observed (*P* < 0.001, MAE = 18.20, RMSE = 25.22).

**Table 3:** Test of regression equations for body composition prediction using 30% of DEXA measurements and chemical analysis of Trial 1 data. Significance level (*P*) of regression between DEXA estimates and corresponding chemical analysis values. Coefficient of determination (R^2^), mean absolute error (MAE) and root-mean-squared error (RMSE) are shown (*n* = 88).

Item	*P*	R^2^	MAE	RMSE
Weight, g	<0.001	0.999	18.20	25.22
Fat, %	<0.001	0.781	0.93	1.21
Fat, g	<0.001	0.984	13.07	18.33
Ash, %	-	-	-	-
Ash, g	<0.001	0.947	3.91	5.60
SLT^1^, %	<0.001	0.566	1.08	1.31
Water, %	<0.001	0.533	1.15	1.42
Protein, %	-	-	-	-
SLT^1^, g	<0.001	0.998	29.66	40.46
Water, g	<0.001	0.997	26.60	37.55
Protein, g	<0.001	0.991	10.95	15.32

1 SLT: soft lean tissue.

**Figure 3:**
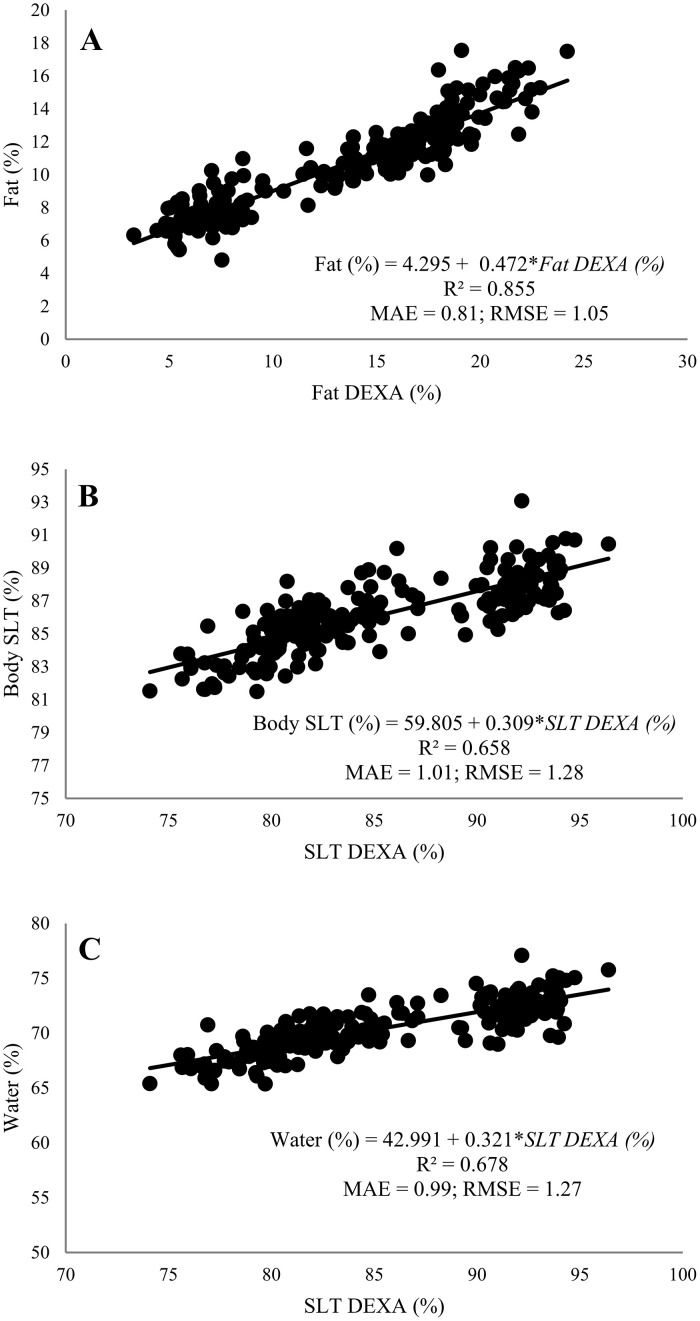
Least-squares regressions developed using 70% of Trial 1 dataset for prediction of contents of broiler body composition: fat (A), soft lean tissue (SLT) (B), and water (C). The DEXA measurements are independent variables on *x*-axis and corresponding chemical analysis values are dependent variables on *y*-axis. Regression equations, coefficients of determination (R^2^), mean absolute error (MAE) and the root-mean-squared error (RMSE) are shown in every graphic (*n* = 206).

For both fat content and fat mass, *β*_0_ and *β*_1_ were significant (*P* < 0.001) and a high positive correlation was observed, with the R^2^ value of parameter verification for fat content at 0.833 and for Fat mass 0.982 (Table 2). The linear regression equations developed to estimate broiler fat content (R^2^ = 0.855, MAE = 0.81, RMSE = 1.05) and fat mass (R^2^ = 0.981, MAE = 13.87, RMSE = 21.28) are shown in Figures 3A and 2B, respectively. The test of the regression equations of fat content (R^2^ = 0.781, *P* < 0.001, MAE = 0.93, RMSE = 1.21) and fat mass (R^2^ = 0.984, *P* < 0.001, MAE = 13.07, RMSE = 18.33) with the Trial 1 data split is shown in Table 3. The ash content's *β*_0_ and *β*_1_ were significant (*P* < 0.001); nevertheless, the R^2^ value was low (0.070) and a negative correlation (*ρ* = −0.27) was observed (Table 2). Based on these results, a linear regression equation for ash content was not developed. For ash mass, the *β*_0_ and *β*_1_ were also significant (*P* < 0.001), and a high positive correlation (*ρ* = 0.98) and R^2^ value (0.953) was observed (Table 2). The linear regression equation developed for ash mass (R^2^ = 0.956, MAE = 3.98, RMSE = 5.61) is also shown in Figure 2C. The test of linear regression equations using the Trial 1 data split for ash mass (R^2^ = 0.947, *P* < 0.001, MAE = 3.91, RMSE = 5.60) is shown in Table 3.

The SLT content and SLT mass were compared to the sum of water and protein obtained through humidity (via dry matter) and wet crude protein chemical analysis. *β*_0_ and *β*_1_ were significant (*P* < 0.001) for both SLT content and SLT mass; therefore, a positive correlation (*ρ* = 0.79) was observed for SLT content (R^2^ = 0.628) and a high positive correlation (*ρ* = 1) was observed for SLT mass (R^2^ = 0.997) (Table 2). A linear regression equation was developed for SLT content (R^2^ = 0.658, MAE = 1.01, RMSE = 1.28) and for SLT mass (R^2^ = 0.997, MAE = 35.73, RMSE = 52.45) and is shown in Figures 3B and 2D, respectively. The test of regression equations of SLT content (R^2^ = 0.566, *P* < 0.001, MAE = 1.08, RMSE = 1.31) and SLT mass (R^2^ = 0.998, *P* < 0.001, MAE = 29.66, RMSE = 40.46) with Trial 1 data split is shown in Table 3. As SLT measured by the DEXA scanner represented the muscle tissue composed of protein plus water, the SLT was considered to be the counterpart of chemical-based water and protein data for the linear regression equations development. Considering this, for water content and water mass, both *β*_0_ and *β*_1_ were significant (*P* < 0.001) and a positive correlation (*ρ* = 0.80) for water content (R^2^ = 0.635) and a high positive correlation (*ρ* = 1) for water mass (R^2^ = 0.997) was observed (Table 2). A linear regression equation was also developed for water content (R^2^ = 0.678, MAE = 0.99, RMSE = 1.27) and for water mass (R^2^ = 0.997, MAE = 29.56, RMSE = 43.94) and is shown in Figures 3C and 2E, respectively. The test of regression equations of water content (R^2^ = 0.532, *P* < 0.001, MAE = 1.15, RMSE = 1.42) and water mass (R^2^ = 0.997, *P* < 0.001, MAE = 26.60, RMSE = 37.55) with Trial 1 data is shown in Table 3. Regarding protein content, *β*_0_ was significant (*P* < 0.001) but *β*_1_ was not significant (*P* = 0.22) and no correlation between DEXA measurements and chemical data was observed (Table 2). Based on these results, a linear regression equation for protein content was not developed. However, in relation to protein mass, both *β*_0_ and *β*_1_ were significant (*P* < 0.001) and a high positive correlation (*ρ* = 0.99) and R^2^ value (0.989) was observed (Table 2). The linear regression equation developed to estimate broiler protein mass (R^2^ = 0.989, MAE = 12.94, RMSE = 19.05) is shown in Figure 2F. The test of linear regression equation using Trial 1 data for protein mass (R^2^ = 0.991, *P* < 0.001, MAE = 10.95, RMSE = 15.32) is shown in Table 3.

### Trial 2: Linear Regression Equations Validation

**Figure 4:**
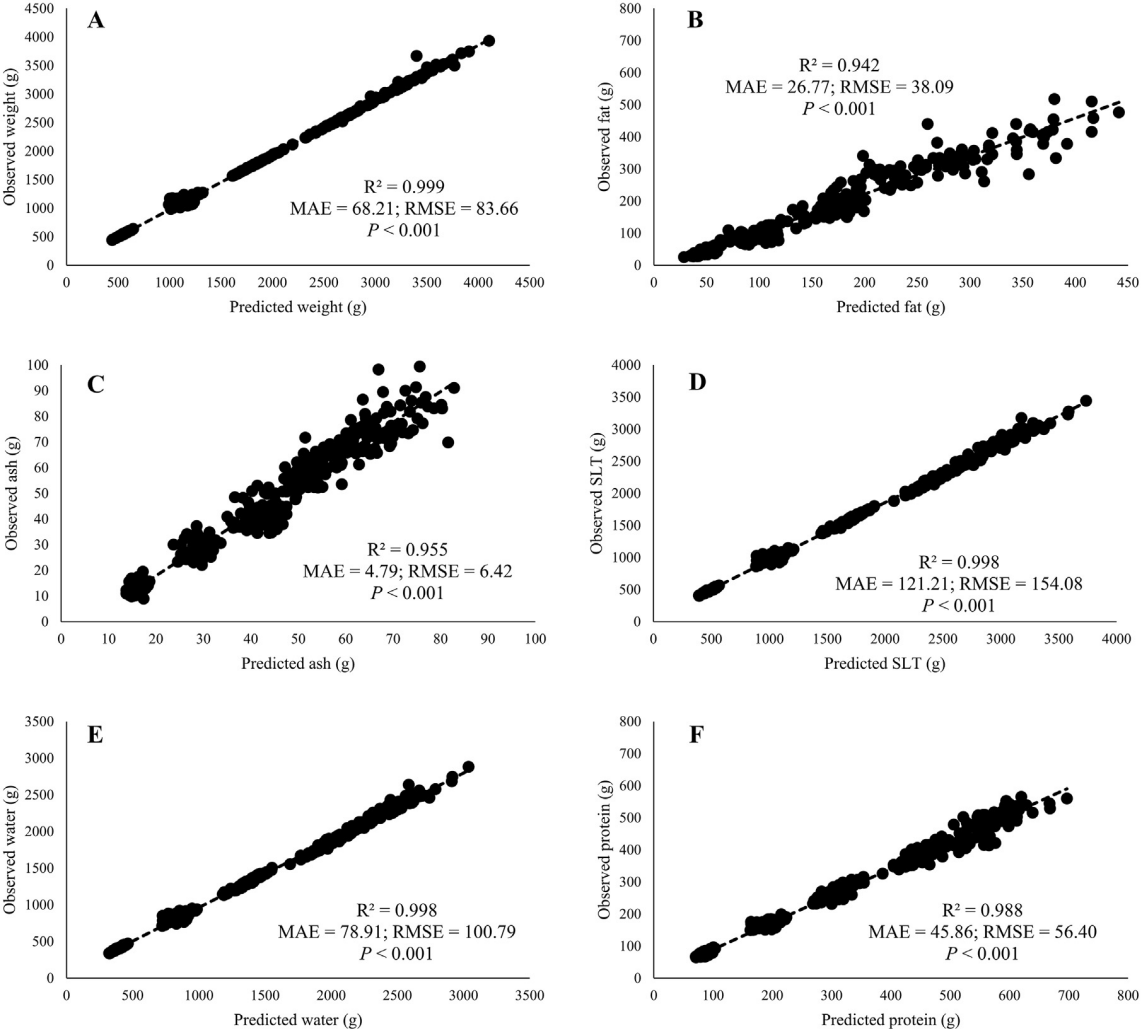
Validation with external data of least-squares regressions using DEXA for prediction of weights of broiler body composition: weight (A), fat (B), ash (C), soft lean tissue (SLT) (D), water (E), and protein (F). The predicted measurements are independent variables on *x*-axis and corresponding observed chemical values are dependent variables on *y*-axis. Coefficients of determination (R^2^), mean absolute error (MAE), the root-mean-squared error (RMSE) and *P* are shown in every graphic (*n* = 385, except fat *n* = 275).

**Figure 5:**
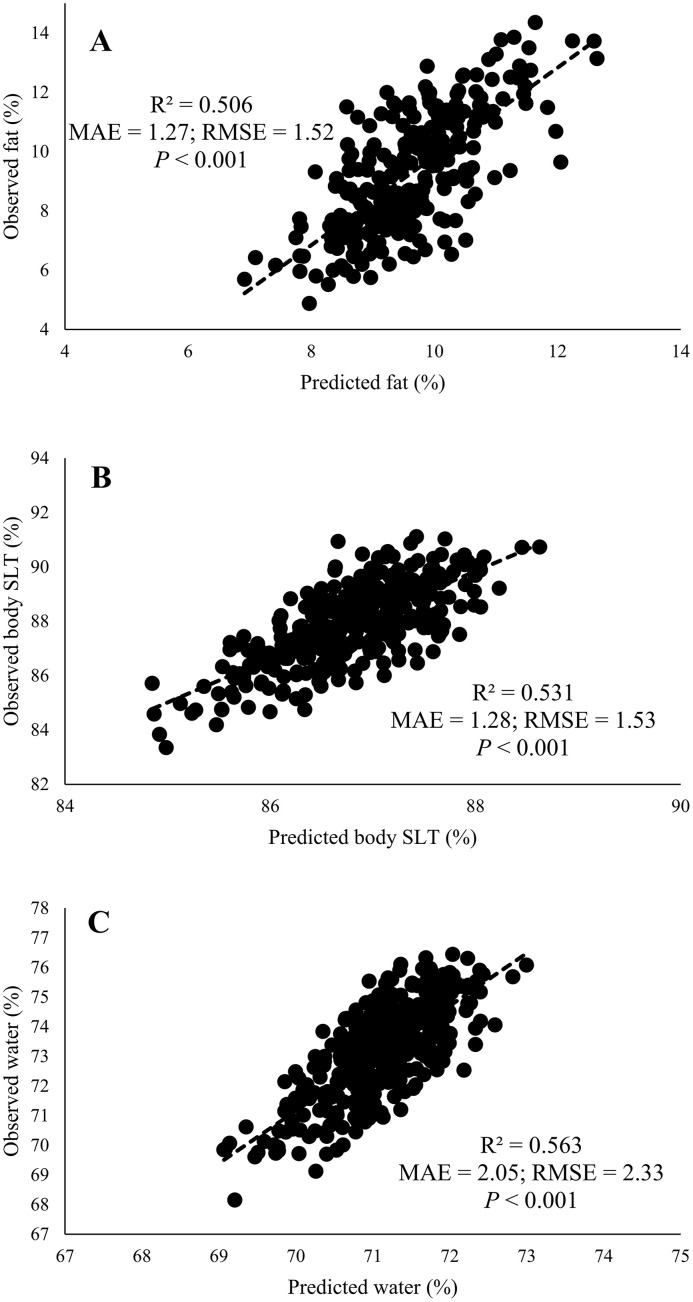
Validation with external data of least-squares regressions using DEXA for prediction of contents of broiler body composition: fat (A), soft lean tissue (SLT) (B), and water (C). The predicted measurements are independent variables on *x*-axis and corresponding observed chemical values are dependent variables on *y*-axis. Coefficients of determination (R^2^), mean absolute error (MAE), the root-mean-squared error (RMSE) and *P* are shown in every graphic (*n* = 385, except fat *n* = 275).

The linear regression equations validation was performed by comparing the values estimated using the equations developed in Trial 1 (predicted values) against the values observed via chemical analysis through a regression. For all variables expressed in weight, high R^2^ values were observed (>90%), whereas, for all variables expressed in content, the R^2^ values observed were around 50%. The regressions of predicted vs. observed values for each variable expressed in weights and contents are shown in Figure 4, Figure 5, respectively.

## DISCUSSION

The first Trial aimed to compare the DEXA scanner estimates against chemical carcass analysis of broilers aged 1 to 42 d and to establish reliable predictive linear regression equations. Chemical analysis is still the gold standard for measuring chicken broiler composition, and consequently, DEXA accuracy can be determined by comparison (Schallier et al., 2019). Furthermore, Trial 2 evaluated the validation of prediction equations developed in Trial 1 using external data. Validation is highly recommended for verifying whether a mathematical model is useful and accurate to a real system (Inca et al., 2022).

Independent of the method used to measure the FFB composition, DEXA and chemical analysis estimates showed similar tissue pattern of variation in the following (descending) order: fat, ash, protein, weight, SLT, and water. The diets and age conditions applied to the animals in Trials 1 and 2 successfully provided a diverse dataset for application of tests, development of prediction equations, and subsequent validation.

The results of development of the linear regression equations (Trial 1) demonstrated that DEXA was an effective approach for predicting body carcass composition of broilers. The weight determined by DEXA as the sum of SLT mass, fat mass, and BMC showed an almost perfect correlation with scale weight in this study. The equation slope of 1.079 demonstrated that DEXA slightly underestimated the FFB weight of chickens, which may be due to underestimation of SLT mass and ash and the overestimation of fat, which is consistent with the observations of Schallier et al. (2019). Nonetheless, other studies with chickens demonstrated an overestimation of DEXA weight even in the presence of a high positive correlation with scale weight (i.e., Mitchell et al., 1997; Swennen et al., 2004; Salas et al., 2012; Gonçalves et al., 2018; Martinez et al., 2022b). This inconsistency among previous studies, although small, reinforces the importance of calibration of specific instruments, software versions, and ROI methodologies. The criteria for stating equation robustness were to observe high R^2^ and low MAE or RMSE error metrics (Martinez et al., 2022b). Therefore, the linear equation for weight demonstrated a high accuracy when we observed the reduction of 6.92 g MAE (or, RMSE = 13.77) value of testing using Trial 1 split data.

The high correlation of fat mass observed in this study is also in agreement with what was observed (*ρ* = 0.98) by Schallier et al. (2019). Furthermore, the R^2^ (98%) of this study was higher and was corroborated by other values in the literature ranging from 89 to 96% (Korine et al., 2004; Swennen et al., 2004; Salas et al., 2012; Gonçalves et al., 2018; Schallier et al., 2019). An exception to this were the results of Mitchell et al. (1997), who observed a value of 62% for R^2^ that the authors attributed to the DEXA scanning mode, program version, and to the weight of the birds, as birds weighing less than 2,000 g had more discrepancies in fat mass. However, Gonçalves et al. (2018), even with a high R^2^ (91%), also observed similar discrepancies in birds with low body weight (7 d of age), which had high fat mass; the contrary occurred in heavyweight birds (28 and 77 d of age). In the present study, DEXA overestimated fat mass (*β*_1_ = 0.756), which is in agreement with other studies (Korine et al., 2004; Swennen et al., 2004; Salas et al., 2012; Schallier et al., 2019). This was previously attributed to variation in soft tissue hydration, as discussed by Pietrobelli et al. (1998), although the authors concluded in the same work that the magnitude of DEXA fat estimation error related to soft tissue hydration is small under normal circumstances, and should not pose any substantial limitations to the accuracy of DEXA technique. Nonetheless, Korine et al. (2004) pointed out the following reasons for DEXA fat overestimation in their study: first, DEXA calibration was based on rat bone density (mouse phantom of Lunar PIXImus 2 model); second, the feathers were mistaken as fat by the DEXA scan; lastly, the ether petroleum used as a solvent for lipid extraction extracted mainly triglycerides (84% of total lipids), whereas the DEXA scanner estimates total lipids. In the present study, the first point can be disregarded due to this DEXA scanner model's and software differences. Although defeathered chickens were used in this study, it is important to note that, while Mitchell et al. (2011) stated that chicken feathers are not detected by DEXA, the study by Korine et al. (2004) focused on migratory birds that may have very different feather compositions than domestic broiler chickens. In this context, the last point about lipid extraction method may explain the fat mass overestimation observed in our study. Another topic discussed by the authors is that DEXA's X-ray attenuation coefficient distinguishes a pixel based on the coefficients of high (e.g., bone tissue) and low (e.g., soft tissue) attenuation energy levels; in areas close to the bones that may not contain adequate amounts of soft tissue, such as the wings, neck, head, and feet, the interpolation process is necessary to estimate the soft tissue composition. In such cases, the process is based on reconstruction of the soft tissue composition by applying the average body fat percentage of the entire animal; thus, if this percentage is greater than the fat percentage of these areas, the fat will be overestimated (Mitchell et al., 1997; Nagy and Clair, 2000; Korine et al., 2004). Regarding fat content, there was also a positive correlation (*ρ* = 0.91) between DEXA and chemical analysis results, while slightly less than that observed for fat mass. Although this decrease is in agreement with other works, the correlation found in our study was higher compared to Swennen et al. (2004) and Schallier et al. (2019), who found correlation values 0.59 and 0.77, respectively. These differences may be related to evolution of DEXA software, which has been calibrated over the years; additionally, the scanner used in this study was more recently developed. The linear equation for fat mass proved to be robust in the split data Trial 1 test, as no changes in R^2^ and a slight reduction of MAE and RMSE values were observed. However, the fat content prediction equation test showed a reduction of 7.43% in the R^2^ value and an increase of 0.12% in MAE (or, RMSE = 0.16) value, indicating a possible inaccuracy of this prediction model.

Ash content was compared using the DEXA BMC percentage as an independent variable, and as result, a negative correlation and a small R^2^ value were observed in this study, thereby discarding the possibility of prediction model development and corroborating results observed in other studies (Swennen et al., 2004; Schallier et al., 2019). A possible explanation for these results is that ash content comprises a small range of values among all age groups of chicken, reaching 0.3 to 1.6% for BMC (fold difference of 5.3) and 2 to 3% for chemical ash (fold difference of 1.5). On this basis, a linear regression model, contrasting ash chemical and BMC content, with a low coefficient of determination, was expected. However, a high correlation was observed for ash mass, as well as reported by Swennen et al. (2004) and Schallier et al. (2019). A high R^2^ was likewise observed by these authors, as well as by Salas et al. (2012) and Gonçalves et al. (2018). Only 1 study observed a low coefficient of determination of 46% between ash mass and DEXA BMC, and the authors were unable to clarify the absence of correlation (Mitchell et al., 1997). Observing the equation slope (*β*_1_ = 1.578), DEXA greatly underestimated the ash quantity of broiler bodies compared to chemical ash weight. This finding was also reported in other studies (Swennen et al., 2004; Salas et al., 2012; Gonçalves et al., 2018; Schallier et al., 2019). The observed discrepancy between BMC and ash chemical weight may be attributed to the fact that DEXA exclusively measures the mineral content of bones; conversely, mineral chemical analysis, carried out by burning the sample in a muffle furnace, takes into account the entire broiler body, including bones and nonbone tissues, which also contain substantial mineral content (such as organs, muscle, and liver) (Johnson et al., 2016; Gonçalves et al., 2018). Additionally, based on the results of the linear regression test using split data of Trial 1, DEXA BMC allowed a good estimation of total body ash mass of broilers when corrected with the corresponding linear regression. A slight reduction of 0.92% in R^2^ was observed, whereas the MAE value was reduced to 0.07 g (or, RMSE = 0.01), indicating the robustness of the prediction model.

Water comprises more than 70% of the whole bodyweight of broilers. Because bone and (hydrophobic) fat have less water content due to their chemical nature, it is assumed that SLT is composed of protein and a larger proportion of water (Brommage, 2003). Hence, the comparison between DEXA SLT and water plus protein obtained chemically was accepted as “chemical soft lean tissue mass.” In this study, a high positive correlation between DEXA SLT mass and chemical SLT, water, or protein mass was consistent with previous works on chickens (Mitchell et al., 1997; Swennen et al., 2004; Salas et al., 2012; Gonçalves et al., 2018; Schallier et al., 2019). In spite of this, an overestimation was observed by Swennen et al. (2004) in SLT determined by DEXA, and the same was found by Gonçalves et al. (2018) in chickens with heavy weights. On the other hand, similar to the present study (*β*_1_ = 1.157), Schallier et al. (2019) observed DEXA underestimated the SLT mass (*β*_1_ = 1.047), which the authors attribute to characteristics of every unique scanner and software, emphasizing the need for proper calibration. Furthermore, the linear regression developed with R^2^ = 0.997 and MAE = 35.73 g (RMSE = 52.45) allowed a proper SLT prediction based on DEXA estimations; as when tested with Trial 1 split data, a reduction of 6.07 g in MAE (or, RMSE = 11.99) was observed. The same behavior was observed when linear equations for water and protein mass were tested using Trial 1 split data, as reductions in MAE of 2.96 g (or, RMSE = 6.39) and 1.99 g (or, RMSE = 3.73) were observed, respectively.

Similarly to fat content, SLT (*ρ* = 0.79) and water (*ρ* = 0.80) content showed a positive correlation to weight but to a lesser extent (*ρ* = 1, for both). In relation to protein content, no correlation was observed, making it impossible to develop a prediction equation based on DEXA SLT estimates. Although diets guaranteed a body protein content variation (**CV** ∼5%) within the broiler ages, the protein content percentages among the different age groups were 15.55% (1 d), 15.56% (14 d), 15.95% (28 d), and 15.80 % (42 d), with practically no variations between the group ages. When compared to protein mass, we observed among the same different age groups, results of 6.91 g (1 d), 74.96 g (14 d), 243.5 g (28 d), and 479.4 g (42 d); this explains why no significance was observed for *β*_1_ of protein content percentages but was observed for protein mass. These results were similarly observed in other studies involving chickens, where the correlation between DEXA values and the water content was better than that for the protein (Mitchell et al., 1997; Schallier et al., 2019). These observations can be attributed to the high content of water relative to protein within the tissue when calculating their relationship with SLT. The equations developed for SLT and water content showed R^2^ values of 66 and 68%, respectively, which is not high, and can cause inaccurate prediction. Therefore, the equations tests using Trial 1 split data demonstrate a reduction in R^2^ of 9.22% for SLT content and 14.52% for water content, as well as an increase in MAE value of 0.07 and 0.16%, respectively. Consequently, accurately distinguishing minor variations in fat, SLT, and water content within a narrow range of tissue weights is more difficult (Schallier et al., 2019). Thus, a possible explanation for the results observed for the variables, expressed as percentage of fat, water, SLT, and especially for ash and protein, can be attributed to the smaller range of values (0–100%, as a ratio of total body weight) compared to the wide range of absolute data points (Swennen et al., 2004). Nevertheless, validation with external data is still necessary to ensure model efficiency and no overfitting (Martinez et al., 2022c).

The method of validation using unavailable data when the model was developed is considered the most rigorous approach (Harrell, 2015), and it has been demonstrated that this method can identify high prediction errors in models previously considered accurate at the development phase (Inca et al., 2022; Martinez et al., 2022c). In this sense, Trial 2 was conducted to obtain external data and use it for validation of the prediction equations developed in Trial 1. The criteria for validation were the same as those described by Martinez et al. (2022c), which considered a conservative change for external validation if the ∆R^2^ < 25% (∆R^2^ = R^2^ of prediction model ˗ R^2^ of validation), when the R^2^ is high enough. In this way, the equations for prediction of broiler FFB weight, fat mass, ash mass, SLT mass, water mass, and protein mass based on DEXA estimates were validated (all regressions showed ∆R^2^ = 0, except for fat mass, for which ∆R^2^ = 4%) and can be securely used as a tool to measure chicken body composition. An increase in the MAE and RMSE values were observed for all of these variables in validation, although the opposite was observed in the results of the Trial 1 split-data test. The reason for this is that splitting data may cause an overestimate of the efficiency of models, underlining the need for validation using external data (Harrell, 2015; Martinez et al., 2022c). In relation to fat content, the linear regression for prediction was not validated (∆R^2^ = 35%), and the R^2^ between the predicted and real observations was considered low for this purpose (R^2^ = 51%). Additionally, SLT and water content showed ∆R^2^ values of 13 and 12%, respectively. However, the coefficient of determination for both was low, with values of 53 and 56%, respectively. Based on these results, the prediction equations for any variable expressed as a percentage in this study were not validated, indicating that DEXA is only recommended to estimate the absolute weight of body tissues in broilers. Hence, different model structures to predict these tissue percentages may be explored. Nonetheless, other models validated herein can be used to obtain the percentage of the target tissue as ratio of total weight (e.g., ash content = ash mass/weight × 100).

It should be noted that, when DEXA estimates of 1-day-old birds from Trial 2 were applied in the prediction equations, the body prediction results were not reliable, especially for the protein mass-negative values that were observed. Because of this, we decided not to use these records for validation. A possible explanation for these results could be one disadvantage of GE Prodigy DEXA, which is the lower weight limit of approximately 250 g (Johnson et al., 2016). Despite this lower limit of DEXA, one aim of this study was to test the accuracy of measurements at this early stage, as well as to obtain a wide range of dependent variable for prediction equations, similar to Salas et al. (2012). Another disadvantage mentioned in studies that use DEXA to evaluate the body growth rate of chickens, is that a parallel study using the slaughter technique must be carried out to evaluate the feather growth rate (Gonçalves et al., 2018; Alves et al., 2019). Due to the inability of DEXA to detect chicken feathers, it is impossible to measure the composition of feathers growth of individuals over time (Gonçalves et al., 2020). However, for the meat industry it would not be a problem, as one of the market's interests is the body composition of the FFB carcass, mainly protein and fat.

In the current study, only male broilers were used for Trials 1 and 2. However, the prediction models obtained here can be reliably used for female birds as well, as no trait associated with sex influence on the DEXA measurements has been previously reported in the literature (Salas et al., 2012; Soladoye et al., 2016; Gonçalves et al., 2018). The strain of bird also may not have an influence on DEXA results, as Swennen et al. (2004) developed the prediction equations with the Cobb strain and validated it with the Ross strain. Therefore, further studies evaluating the effect of different chicken genetic strains on DEXA-traits are suggested, as was observed for swine (Soladoye et al., 2016).

In the literature, there are several studies which could benefit from the equations developed here. Castro et al. (2019), who studied the effect of L-arginine supplementation, used the DEXA scanner (Prodigy, GE Healthcare) method to evaluate the body composition of broiler chickens. When applying the equations of our study, in addition to properly calibrating the data, the authors would discover that increasing L-arginine could result in an increasing protein weight of broilers, which would require statistical procedures to verify significance. Wang et al. (2019), investigating the efficacy of different levels of phytase and multicarbohydrase on growth performance and bone mineralization in male broilers, could take advantage of our prediction equations to evaluate the effect of body composition on the body weight gain, since the authors used DEXA scanner but did not used the DEXA results of SLT. In a study by Chen et al. (2020), although the authors did not specify that they used GE Healthcare DEXA scanner and used the small animal module, the use of DEXA results in the prediction equation could contribute to enriching the understanding of the effect of 25-hydroxyvitamin D_3_ supplementation for laying hens on the animals’ total body compositions, most notably on total body ash, in addition to bone structural development. For broiler breeders, caution should be exercised when using the prediction equations developed in our study. Studies employing DEXA to estimate body composition (Aranibar et al., 2020; Avila et al., 2023), could benefit from these prediction equations for accurate calibration of body composition. Nevertheless, researchers must carefully consider poultry age and body traits before applying prediction equations to avoid extrapolating results.

In summary, we demonstrate here that DEXA is a good method of measuring body carcass composition of broiler chickens. Furthermore, DEXA, in contrast to chemical analysis, is a nondestructive and noninvasive method that allows longitudinal studies to be carried out using a limited number of birds, as there is no need to kill the animals. It is also important to emphasize that chemical analysis, although it remains the gold standard, is associated with increased labor, time consumption, multiple reagents and machinery usage, higher costs, and the generation of pollution residuals. In the poultry industry, dual X-ray technology can be used for meat inspection, quality control, and to typify carcasses. Thus, predictive equations, such as those validated in the present study, are strongly recommended for proper calibration of DEXA estimates.

## CONCLUSIONS

The linear models for chicken body composition prediction validated in this study are possibly specific to particular equipment, software version, and procedures (such as bird positioning and ROI specifications). Nonetheless, the same equation structures can be adapted to new data using different DEXA scanner systems by following the same development and validation processes of this study to acquire new customized prediction models. In Trial 1, predictive linear models were developed for broiler weight, fat mass, ash mass, SLT mass, water mass, protein mass, fat content, SLT content, and water content. Therefore, Trial 2 was conducted for validation, and remarkably, all absolute tissues were validated, indicating a high accuracy prediction of these equations. Thus, the prediction equations validated, can be used to obtain the tissue content as ratio of total weight. The DEXA method is an effective approach to measuring the body composition of broilers by using regression equations to calibrate results. The equations developed in this study could greatly contribute to DEXA applicability in poultry research, genetic improvement, meat production, and the poultry industry.

## Appendix Supplementary materials

Image, application 1
