# Supplementary material for: Dual-energy X-ray absorptiometry: an effective approach for predicting broiler chicken body composition
Source: Poult Sci. 2023 Dec 9;103(2):103363. doi: 10.1016/j.psj.2023.103363 (PMC10788280; doi:10.1016/j.psj.2023.103363)
Supplement: Supplementary file 1 [file mmc1.docx]

Table S1. Experimental diets of Trial 1. In a basal diet, using DL-Met or DL-2-hydroxy-4-methylthio butanoic acid (HMTBA) as source, increasing levels (in parenthesis) of Met was supplemented in substitution of starch. A plus diet with high DL-Met supplementation and more Biolys was also formulated. Totalizing 14 experimental diets.

| Ingredients (%) | 1 - 14 days | 14 - 28 days | 28 - 42 days |
| --- | --- | --- | --- |
|  | Initial | Grower | Finisher |
| Corn | 53.32 | 61.20 | 59.61 |
| Soybean meal, 46% | 32.05 | 25.55 | 27.56 |
| Soy Concentrate, 62% | 6.37 | 4.00 | 0 |
| Starch | (2, 1.96, 1.92, 1.87, 1.8, 1.7, 1.6, 1.34) | (2, 1.96, 1.92, 1.88, 1.83, 1.75, 1.67, 1.47) | (2, 1.96, 1.92, 1.88, 1.83, 1.76, 1.68, 1.48) |
| Soy oil | 2.36 | 3.14 | 6.87 |
| Salt | 0.28 | 0.17 | 0.17 |
| Sodium bicarbonate | 0.15 | 0.49 | 0.56 |
| Dicalcium phosphate | 1.84 | 1.67 | 1.40 |
| Limestone | 0.71 | 0.64 | 0.64 |
| DL – Methionine, 99.7% or HMTBA, 88%^1^ | (0, 0.04, 0.08, 0.13, 0.2, 0.3, 0.4) | (0, 0.04, 0.08, 0.12, 0.17, 0.25, 0.33) | (0, 0.04, 0.08, 0.12, 0.17, 0.24, 0.32) |
| Biolys, 54.6%^2^ | (0.12, 0.38) | (0.22, 0.42) | (0.26, 0.46) |
| L-Threonine, 98% | 0.13 | 0.15 | 0.16 |
| L-Valine | 0.10 | 0.12 | 0.14 |
| L-Isoleucine | 0.04 | 0.10 | 0.10 |
| L-Arginine | 0 | 0.06 | 0.08 |
| L-Glycine | 0.11 | 0.10 | 0.11 |
| Choline chloride, 70% | 0.08 | 0.08 | 0.08 |
| Mineral premix^3^ | 0.13 | 0.12 | 0.09 |
| Vitamin premix^4^ | 0.13 | 0.12 | 0.09 |
| Coccidiostátic^5^ | 0.06 | 0.06 | 0.06 |
| Antioxidant^6^ | 0.02 | 0.02 | 0.02 |
| *Calculated composition*^7^ | | | |
| Metabolizable Energy, kcal/kg | 2974 | 3107 | 3344 |
| Crude protein, % | 23.13 | 19.53 | 17.98 |
| SID Met + Cys, % | (0.6, 0.64, 0.68, 0.73, 0.8, 0.9, 1) | (0.52, 0.56, 0.6, 0.64, 0.69, 0.77, 0.85) | (0.48, 0.52, 0.56, 0.6, 0.65, 0.72, 0.8) |
| SID Methionine, % | (0.31, 0.35, 0.39, 0.44, 0.51, 0.61, 0.71) | (0.27, 0.30, 0.34, 0.38, 0.43, 0.51, 0.59) | (0.25, 0.28, 0.32, 0.36, 0.41, 0.48, 0.55) |
| SID Cysteine, % | 0.29 | 0.26 | 0.24 |
| SID Lysine, % | (1.2, 1.34) | (1.03, 1.14) | (0.96, 1.07) |
| SID Threonine, % | 0.86 | 0.75 | 0.71 |
| SID Tryptophan, % | 0.25 | 0.20 | 0.19 |
| SID Arginine, % | 1.41 | 1.20 | 1.14 |
| SID Valine, % | 1.06 | 0.92 | 0.86 |
| SID Isoleucine, % | 0.91 | 0.81 | 0.76 |
| SID Leucine, % | 1.71 | 1.48 | 1.35 |
| SID Histidine, % | 0.57 | 0.46 | 0.42 |
| SID Phenylalanine, % | 1.01 | 0.84 | 0.77 |
| Sodium, % | 0.16 | 0.21 | 0.23 |
| Non-phytic phosphorus, % | 0.49 | 0.42 | 0.36 |
| ^1^ Thirteen diets: basal diet without any Met supplementation (zero) and six levels of methionine supplementation as percentage (% of Met) using each DL-methionine or HMTBA as Met source; | | | |
| ^2^ One diet with high supplementation of DL-Met also received more Biolys; | | | |
| ^3^ Composition per kg of product: manganese, 58.36 mg; zinc, 54.21 mg; iron, 41.68 mg; copper, 8.31 mg; selenium, 250 mg and iodine 843 mg. | | | |
| ^4^ Composition per kg of product: vit. A, 9,637,000 UI; vit. D3, 2,409,000 UI; vit. E, 36,100 UI; vit. B1, 2.59 g; vit B2, 6.45 g; vit. B6, 3.61 g; pantothenic acid, 12.95 g; vit K3, 1.93 g; Folic acid, 903 mg; niacin, 39.20 g; vit B12, 15.9 mg; biotin, 89.8 mg; | | | |
| ^5^ Salinomycin (12%); | | | |
| ^6^ Hydroxybutyltoluene – BHT; | | | |
| ^7^ Calculated values were based on the ingredient’s nutritional composition (Rostagno et al., 2017). | | | |

Table S2. Experimental diets of Trial 2. In a basal diet, using each inorganic or organic mineral sources, increasing levels (in parenthesis) of Mn, Zn, Cu, Fe and Se were supplemented. A diet without mineral supplementation was also formulated. Totalizing 9 experimental diets.

| Ingredients (%) | 1 - 21 days | 21 - 42 days |
| --- | --- | --- |
|  | Initial/Grower | Finisher |
| Corn | 46 | 54 |
| Soybean meal, 45% | 45 | 37 |
| Soy Oil | 4.53 | 5.28 |
| Salt | 0.53 | 0.49 |
| Dicalcium phosphate | 1.27 | 0.81 |
| Limestone | 0.97 | 0.84 |
| DL-Methioine, 98% | 0.333 | 0.288 |
| L-Lysine HCl, 80% | 0.122 | 0.156 |
| L-Threonine, 98% | 0.056 | 0.058 |
| Choline chlorine, 60% | 0.1 | 0.1 |
| Mn, ppm^1^ | (100, 32.83, 73.98, 53.40, 0) | (100, 25.90, 58.36, 42.13, 0) |
| Zn, ppm^1^ | (80, 30.54, 68.72, 49.63, 0) | (80, 24.09, 54.21, 39.15, 0) |
| Cu, ppm^1^ | (12, 4.58, 10.54, 7.56, 0) | (12, 3.61, 8.31, 5.96, 0) |
| Fe, ppm^1^ | (60, 22.91, 52.84, 37.87, 0) | (60, 18.07, 41.68, 29.88, 0) |
| Se, ppm^1^ | (0.3, 0.138, 0.317, 0.227, 0) | (0.3, 0.109, 0.25, 0.179, 0) |
| Potassium Iodate (I), ppm | (1.1, 0) | (1.1, 0) |
| Vitamin Supplement^2^ | 0.14 | 0.10 |
| Coccidiostatic^3^ | 0.06 | 0.06 |
| Antioxidant^4^ | 0.02 | 0.02 |
| Growth promoter | 0.01 | 0.01 |
| Enzyme^5^ | 0.02 | 0.02 |
| Inert^6^ | 0.84 | 0.77 |
| *Calculated composition*^7^ | | |
| Metabolizable Energy, kcal/kg | 3.066 | 3222 |
| Crude protein, % | 24.60 | 21.60 |
| SID Lysine, % | 1.326 | 1.160 |
| SID Methionine, % | 0.653 | 0.575 |
| SID Met + Cys, % | 0.974 | 0.862 |
| SID Threonine, % | 0.868 | 0.769 |
| SID Tryptophan, % | 0.280 | 0.238 |
| SID Valine, % | 1.029 | 0.900 |
| Calcium, % | 0.942 | 0.755 |
| Total Phosphorus, % | 0.699 | 0.591 |
| Available Phosphorus, % | 0.448 | 0.353 |
| Sodium, % | 0.224 | 0.208 |
| Chlorine, % | 0.213 | 0.202 |
| Potassium, % | 0.941 | 0.814 |
| ^1^ Inorganic and organic sources of: Mn - Manganese sulfate, 31.78% and Bioplex Mn, 16.13%; Zn - Zinc sulfate, 35.23% and Bioplex Zn, 16.96%; Cu - Copper sulfate, 25.1% and Bioplex Cu, 10.26%; Fe - Iron sulfate, 31.17% and Bioplex Fe, 16.03%; Se - Sodium selenite, 45.42% and Sel-Plex, 0.23%; | | |
| ^2^ Composition per kg of product: vit. A, 9,637,000 UI; vit. D3, 2,409,000 UI; vit. E, 36,100 UI; vit. B1, 2.59 g; vit B2, 6.45 g; vit. B6, 3.61 g; pantothenic acid, 12.95 g; vit K3, 1.93 g; Folic acid, 903 mg; niacin, 39.20 g; vit B12, 15.9 mg; biotin, 89.8 mg; | | |
| ^3^ Salinomycin (12%); | | |
| ^4^ Hydroxybutyltoluene – BHT; | | |
| ^5^ Alltech SSF. 200 g / ton; | | |
| ^6^ Tixosil; | | |
| ^7^ Calculated values were based on the ingredient’s nutritional composition (Rostagno et al., 2017). | | |

Table S3. R Script used for statistical analysis.

Trial1=read.table(file.choose(),h=T) ### Entering the dataset of Trial 1, search .txt file in your computer

#Data must be as follows: E.G.

#Trial1 <- data.frame(bird=c(1, 2, 3, 4, 5, n),

# x=c(100, 200, 300, 400, 500, n),

# y=c(110, 190, 320, 405, 499, n))

## Y = chemical analysis values; X = DEXA estimates ##

#Checking the data input

head(Trial1)

Trial1=edit(Trial1) #Editing the dataset if necessary

attach(Trial1)

#Opening packages

library(caret)

library(Metrics)

library(Hmisc)

library(corrplot)

###### First Step - Parameters verification (B0, B1) and correlation#####

model=lm(

y~x,

data = Trial1) #Creating the linear model

summary(model) # verifing the parameters values and p-value

confint(model) # confident interval

res= rcorr(

as.matrix(Trial1),

type = c("pearson")) ## correlation

res

corrplot.mixed(

res$r,

lower = "number",

upper = "square") ## correlation plot

test=cor.mtest(

res$r) #obtaining p-value ($p)

test

###### Step 2 - Prediction equations ##########

#### Spliting data for prediction equations development - creating the data and models for cross validation ####

Amostra =createDataPartition(

Trial1$bird,

p = .70,

list = FALSE) ### spliting the dataset

trein= Trial1[ Amostra,] ## training data [ used for development of prediction equations] -> 70%

teste= Trial1[-Amostra,] ### test data -> 30%

met=trainControl(method = "cv",

number = 5) #creating the anlysis method of Cross validation

set.seed(123)

#Prediction equation

mod= train(y ~ x , data=trein,

method='lm',

trControl = met,

)

print(mod) ### Obtaining the error metrics of equations (RMSE, R^2 e MAE)

mod$finalModel ### assessing the parameters (B0,B1)

dat=cbind(

trein$x,

trein$y) ## Creating columns to use in Excel plot

dat

##### Test of prediction equations##########

### Using the 30 % of data set ####

predictions=predict(

mod$finalModel,

teste) ### calculating the predict based on test data (30%) and using the prediction model developed

model=lm(

teste$y ~ predictions,

data=teste) ## observed vs predict of test

summary(model) ### assessing the R^2

data=cbind(

predictions,

teste$SWg) ## Creating columns to use in Excel plot

data

rmse(teste$y, predictions); mae(teste$y, predictions) ## error metrics

#### Step 3 - Validation using external data ####

###### Trial2 input data (Predicted values were calculated in Excel using the equations developed in Trial1) ########

Trial2=read.table(file.choose(),h=T)

head(Trial2)

Trial2=edit(Trial2)

attach(Trial2)

#Data must be as follows: E.G.

#Trial2 <- data.frame(bird=c(1, 2, 3, 4, 5, n),

# y=c(100, 200, 300, 400, 500, n),

# z=c(110, 190, 320, 405, 499, n))

#y = chemical analysis; z = predicted values ##

##Predict vs observed

modrvsp=lm(

y ~ z,

data=Trial2) ## Observed vs predicted for validation

summary(modrvsp) ### assessing R^2

mae(y, z) # assessing Mean Absolute Error
